# COP30: still failing to meet the challenge

**DOI:** 10.1016/j.lanepe.2025.101559

**Published:** 2025-12-05

**Authors:** The Lancet Regional Health – Europe

A decade after the Paris Agreement was adopted at COP21 in 2015—committing nations to limiting global warming to 1.5 °C above pre-industrial levels—the conclusion of COP30 in Belém, Brazil, on Nov 22, 2025, underscored the persistent failure to meet these commitments. Hosted symbolically in the Amazon, where forests are crucial for global climate stability yet threatened by deforestation and climate change, the summit was expected to signal renewed environmental leadership. Instead, COP30 exposed the widening gap between the urgent measures needed to limit warming to 1.5 °C and the inadequate responses of policy makers.

Since the Paris Agreement was signed, global greenhouse gas emissions have risen by approximately 10%, a stark reminder that international agreements have not led to necessary action. The World Meteorological Organization projects an 80% likelihood that annual average global temperatures will exceed 1.5 °C of warming above pre-industrial levels within 5 years. Approaching this threshold should have galvanised decisive, structural change. Instead, the outcome of COP30 revealed weak ambition in three areas that are crucial to fulfilling climate commitments: fossil fuel transition, adaptation finance, and global health.

The most consequential shortfall was the failure to commit to a fossil fuel phase-out. The final text, named the Mutirão, after the Brazilian tradition of collective community mobilisation for a shared goal, did not live up to its name. 88 countries requested a formal roadmap for the phase-out of coal, oil, and gas, but this was ultimately excluded from the agreement. Consequently, these nations must pursue a fossil fuel transition outside the formal UN process—a clear illustration of the limitations of consensus-based negotiations, which were strained by record opposition, including more than 1600 fossil fuel lobbyists.

The European Union (EU) rejected the initial draft deal, arguing it was too weak to keep the 1.5 °C target within reach. This intervention prevented the adoption of a truly inconsequential outcome and highlighted a deeper problem: the world's four largest emitters, namely the USA, China, India, and Russia, remain largely disengaged. Without their meaningful participation, efforts led by the EU, climate-vulnerable, and high-ambition countries (ie, those advocating for the strongest climate action) cannot achieve the scale of progress needed.

Adaptation finance, which provides funding to help countries prepare for and respond to the impacts of climate change, also fell short. The final agreement included a pledge to so-called Triple Adaptation funding by 2035. Although this pledge is a positive signal, it delays the tripling of adaptation finance to 2035 instead of meeting the 2030 deadline demanded by vulnerable countries. This 5-year gap will leave many regions without the resources they need to cope with escalating climate impacts. Moreover, the pledge also falls short of the widely recognised need of US$300 billion per year for adaptation in developing countries. This financing gap highlights the failure to align commitments with on-the-ground needs.

Efforts to address the health impacts of climate change also saw disappointing results. The Belém Health Action Plan aimed to establish a global framework for climate-resilient health systems, with emphasis on disproportionate impact of climate change on women and Indigenous communities and the need for better surveillance for climate-sensitive diseases, such as dengue and malaria. Yet few countries formally endorsed the plan, and governments offered no direct funding. Instead, the initiative will rely almost entirely on philanthropic support. This dependence on non-state actors underscores a concerning trend in which essential health security is treated as an optional project for private donors rather than a core public responsibility.

Lack of accountability is what allows failures in climate action and health to continue. The UN system excels at collecting evidence and diagnosis problems through reports such as the NDC Synthesis, the Adaptation Gap Report, and the Global Stocktake, but this transparency has not translated into action. Instead, it has created a cycle where underperformance is diligently recorded but rarely remedied. COP30 demonstrated that reporting is insufficient when political leaders are not compelled to act.

The Loss and Damage Fund, first operationalised at COP28, illustrates this inertia. Despite its promise, the fund has received minimal new contributions. Vulnerable nations, constrained by debt and high interest rates, cannot finance climate recovery unaided, while donor governments and major emitters have failed to deliver predictable, grant-based support.

The outcome in Belém shows that a weak and unambitious strategy is not merely a diplomatic shortfall; it is a deliberate policy choice, one that heightens climate and health risks for all. COP30 also underscored the divide between nations committed to stronger climate action and those continuing to resist change which is a deeply concerning signal for the world. This lack of unity undermines hope for the collective effort needed to address the climate crisis. As the world looks to COP31 in Antalya, Türkiye, the window for meaningful intervention is narrowing rapidly.

